# Involvement of Phospholipase C in Photosynthesis and Growth of Maize Seedlings

**DOI:** 10.3390/genes13061011

**Published:** 2022-06-03

**Authors:** Yulei Wei, Xinyu Liu, Shengnan Ge, Haiyang Zhang, Xinyang Che, Shiyuan Liu, Debin Liu, Huixin Li, Xinru Gu, Lin He, Zuotong Li, Jingyu Xu

**Affiliations:** 1 Key Laboratory of Modern Agricultural Cultivation and Crop Germplasm Improvement of Heilongjiang Province, College of Agriculture, Heilongjiang Bayi Agricultural University, 5 Xinfeng Road, Daqing 163319, China; wyl8390@163.com (Y.W.); liuxinyu_20@163.com (X.L.); geshnan@163.com (S.G.); zhanghaiyang_betty@163.com (H.Z.); chexinyang668@163.com (X.C.); liushiyuan55@163.com (S.L.); liudebin1234@126.com (D.L.); lihuixin0318@163.com (H.L.); guxinru2022@163.com (X.G.); linlinhe65@sina.com (L.H.); 2 National Coarse Cereals Engineering Research Center, Heilongjiang Bayi Agricultural University, 5 Xinfeng Road, Daqing 163319, China

**Keywords:** maize (*Zea mays*), phospholipase C, neomycin sulfate, photosynthesis, growth and development

## Abstract

Phospholipase C is an enzyme that catalyzes the hydrolysis of glycerophospholipids and can be classified as phosphoinositide-specific PLC (PI-PLC) and non-specific PLC (NPC), depending on its hydrolytic substrate. In maize, the function of phospholipase C has not been well characterized. In this study, the phospholipase C inhibitor neomycin sulfate (NS, 100 mM) was applied to maize seedlings to investigate the function of maize PLC. Under the treatment of neomycin sulfate, the growth and development of maize seedlings were impaired, and the leaves were gradually etiolated and wilted. The analysis of physiological and biochemical parameters revealed that inhibition of phospholipase C affected photosynthesis, photosynthetic pigment accumulation, carbon metabolism and the stability of the cell membrane. High-throughput RNA-seq was conducted, and differentially expressed genes (DEGS) were found significantly enriched in photosynthesis and carbon metabolism pathways. When phospholipase C activity was inhibited, the expression of genes related to photosynthetic pigment accumulation was decreased, which led to lowered chlorophyll. Most of the genes related to PSI, PSII and TCA cycles were down-regulated and the net photosynthesis was decreased. Meanwhile, genes related to starch and sucrose metabolism, the pentose phosphate pathway and the glycolysis/gluconeogenesis pathway were up-regulated, which explained the reduction of starch and total soluble sugar content in the leaves of maize seedlings. These findings suggest that phospholipase C plays a key role in photosynthesis and the growth and development of maize seedlings.

## 1. Introduction

In Heilongjiang province in northeast China, maize is the major crop and accounts for 30% of the country’s maize production [1]. Compared with grain crops such as rice and wheat, maize has a stronger abiotic stress tolerance and better environmental adaptability. Maize is an important source of feed for livestock and in aquaculture, and is an indispensable raw material used for food, used in medical and health care and used in the chemical industry [2].

Phospholipases are a class of enzymes that hydrolyze glycerophospholipids and can be divided into four categories according to their catalytic substrates, namely, phospholipase A (PLA), phospholipase B (PLB), phospholipase C (PLC), and phospholipase D (PLD) [3]. In animals, phospholipase C selectively catalyzes the hydrolysis of phosphatidylinositol 4,5-bisphosphate (PIP2) [4]. The reaction produces two secondary messengers: diacylglycerol (DAG) and inositol triphosphate (IP3). DAG regulates the activity of a variety of enzymes and structural proteins by binding to the phospholipase C conserved structural domain C2, whereas IP3 binds to and controls Ca^2+^ channels in the endoplasmic reticulum to modulate intracellular Ca^2+^ concentration. Ca^2+^ acts as a secondary messenger and further regulates cellular activity through a complex signaling network [4].

Similar to their animal homologs, plant phospholipase C proteins play vital regulatory roles in many cellular processes [5]. The most significant difference between plant phospholipase C and animal phospholipase C is that plant phospholipase C can be further subdivided into phosphoinositide-specific PLC (PI -PLC) and non-specific phospholipase C (NPC), which is capable of hydrolyzing phosphatidylcholine (PC), phosphatidylethanolamine (PE) and phosphatidylserine (PS) [6,7]. Owing to their unique catalytic properties, plant phospholipase C generates the secondary messengers phosphatidic acid (PA) and hexakisphosphate (IP6), which are involved in various signaling pathways [6,7].

In *Arabidopsis thaliana*, nine PI-PLC and six NPC genes were identified, whereas in rice, the number of genes in the two subfamilies was four and five, respectively [8,9]. Recent studies have shown that phospholipase C (PLC2) affects the polar transport of PIN2 in the *Arabidopsis* root system, resulting in uneven distribution of IAA, and leading to shorter primary roots and inhibition of root hair growth in *Arabidopsis* [10]. Over-expression of *PLC5* inhibited the production of PIP and PIP2, resulting in reduced primary and secondary root production and impaired root hair development. At the same time, the drought tolerance of *Arabidopsis* was increased due to the reduction of stomatal aperture [11]. It has been shown that when *Arabidopsis* was treated with a phospholipase C inhibitor, U73122 or neomycin, the root development was hindered, root length was shortened, lateral roots were reduced and root morphology was disrupted [12]. Over-expression of *PLC3* had no effect on *Arabidopsis* root architecture or seed germination, but it increased plant drought tolerance [13].

In plants, most studies on phospholipase C have focused on the model plant *Arabidopsis* and fewer studies are conducted on field crops. In this study, in order to clarify the roles of phospholipase C in maize growth and development, maize seedlings were treated with phospholipase C inhibitor neomycin sulphate (NS,100 mM), and the effects of the PLC inhibition on the growth and development of maize seedlings were investigated.

## 2. Materials and Methods

### 2.1. Plant Growth, Handling and Sampling

The maize inbred He344 was used in this study. Maize seeds of the same size were selected and disinfected with 10% NaClO for 30 min. After repeated washing with distilled water, the seeds were placed in an incubator at 25 °C for dark germination. After germination, maize seedlings were grown in a growth chamber with 1/2 Hoagland’s nutrient solution (pH = 5.5). The temperature of the growth chamber was set at (25 ± 2) °C/(20 ± 2) °C, with a photoperiod of 16 h/8 h (light/dark) and 60–80% humidity. Two-week-old maize seedlings were treated with 100 mM of neomycin sulphate (NS) for phospholipase C inhibition. Maize leaf samples were collected 1, 2, 3, 5 and 7 d after the NS inhibition treatment. The collected samples were wrapped in tin foil, quickly frozen in liquid nitrogen and stored at −80 °C. At least 3 replicates were applied for each sample [14].

### 2.2. RNA-Seq Analysis and qRT-PCR Validation

Two-week-old maize seedlings were treated with 100 mM of NS and leaf samples (with 3 replicates) were collected 1, 3 and 5 d after the treatment; the non-treated maize samples were used as the control. Total RNA was extracted from leaf tissues of maize seedlings using a TRIzol reagent (Shanghai, China). RNA samples were tested for purity and concentration, and libraries were constructed. After database qualification, RNA sequencing was performed by the Illumina platform, and the data were mapped to maize reference genome B73 RefGen_v3. The RNA-seq data were submitted to the online SRA (sequential read file) database under submission number: SUB9376981.

A differentially expressed genes (DEGs) analysis was performed using a false discovery rate (FDR) of less than 0.01 and |log_2_ fold change| ≥ 1. DEGs were annotated to the Kyoto Encyclopedia of Genes and Genomes (KEGG) (https://www.genome.jp/kegg/, accessed on 18 November 2021) database.

To further validate the reliability of the RNA-seq results, a qRT-PCR analysis was performed. The cDNA was synthesized using ReverTra Ace qPCR RT Master Mix (TOYOBO, Osaka, Japan). Real-time quantitative RT-PCR was performed in 96-well plates using the SYBR Select Master Mix RT-PCR system. *ZmACTIN* and *ZmGAPDH* were used as internal controls. Gene specific primers used for qRT-PCR are listed in Table S1. The results were calculated using the 2^−ΔΔct^ method, and a day 0 maize sample was used as a calibrator.

### 2.3. Phenotypic Analysis of Maize Seedlings under NS Treatment

The phenotype of maize seedlings under NS (100 mM) treatment were photographed and recorded at different time points. The plant height and root length were measured. The roots of the seedlings were separated from the upper part of the roots and the fresh weights of the leaves and roots were measured. The roots were scanned using an Expression 1200XL root system scanner, and the scanned images were analyzed using Win RHIZOTRON software.

### 2.4. Analysis of Photosynthetic Parameters and Chlorophyll Content

Photosynthetic parameters were measured using a Li-6400 XTR photosynthesizer (Li-COR, Lincoln, NE, USA). The light intensity was adjusted to 600 μmol·m^−2^·s^−1^, the temperature was set at 25 °C, the CO_2_ concentration was set at 400 μL·L^−1^ and the light intensities were set at 0 μmol·m^−2^·s^−1^, 100 μmol·m^−2^·s^−1^, 200 μmol·m^−2^·s^−1^, 300 μmol·m^−2^·s^−1^, 400 μmol·m^−2^·s^−1^, 800 μmol·m^−2^·s^−1^, 1000 μmol·m^−2^·s^−1^ and 1200 μmol·m^−2^·s^−1^. The photosynthetic parameters were analyzed by photosynthesis software to simulate photosynthesis, and the ALA and PBG contents were determined according to the previously reported methods [15]. Chlorophyll content was measured as described [16] and anthocyanin content was measured as described [17].

### 2.5. Statistic Analysis

The data were collated using Excel 365, and SPSS 24.0 software was used for one-way ANOVA. Duncan’s test was applied for multiple comparisons and significance of differences analysis. The data were the means of three or more replications, and GraphPad was used for plotting.

## 3. Results

### 3.1. Inhibition of Phospholipase C Activity Affects the Growth and Development of Maize Seedlings

**Figure 1:**
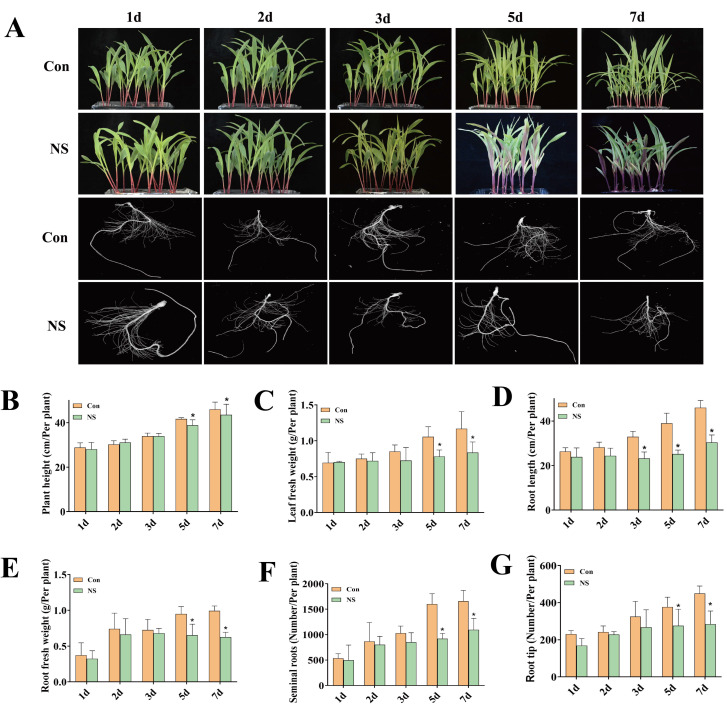
Physiological phenotypes of maize seedlings under phospholipase C inhibition. (**A**), photos of maize seedlings; (**B**), plant height (Con: Control, NS: 100 mM of neomycin sulfate); (**C**), leaf fresh weight; (**D**), root length; (**E**), root fresh weight; (**F**), number of seminal roots; (**G**), number of root tips. Values are shown as mean ± SD (*n*=3 individuals per group). * means that the difference from the control reaches a significant level, *p* < 0.05.

In order to investigate the effects of phospholipase C inhibition on the growth and development of maize seedlings, a 100-mM neomycin sulphate (NS) treatment was carried out on two-week-old maize seedlings. Under NS treatment, as shown in Figure 1A, the leaves of maize seedlings gradually etiolated and wilted in the later stage of treatment (5 d and 7 d) compared with the control. In addition, as can be seen in Figure 1B, the plant height of the NS samples was lower than that of the control group after 5 d of treatment. After 2 days of treatment, the leaf fresh weight of NS samples was lower than that of the control group. The leaf fresh weight decreased by 28.33% in the NS sample at the 7-d time point, and the difference between the NS sample and control reached its maximum (Figure 1C).

The effects of phospholipase C inhibition on root development were visualized (Figure 1A). Under the 100-mM NS treatment, roots of maize seedlings exhibited multiple phenotypes, including reduced root numbers and increased curvature. The root length of maize seedlings decreased significantly after 3 d of NS treatment (Figure 1D). The dry weight of roots was consistently lowered in NS-treated samples compared to the control group, and a maximum reduction of 25.15% in root dry weight was observed on the 7-d time point (Figure 1E). The seminal roots increased during the experiment, but the increase in the NS-treated samples was smaller than in the control, and the difference reached a significant level after 5 d (Figure 1F). The number of root tips increased, and the difference between the treatment and the control reached a significant level after 5 d (Figure 1G).

### 3.2. Transcriptome Analysis of Maize Seedlings under Phospholipase C Inhibition

Based on sequencing by synthesis (SBS) technology and the Illumina high-throughput sequencing platform, 12 RNA samples from leaves of maize seedlings under phospholipase C inhibition (and the non-treated control) were sequenced and assembled, and a total of 86.12 Gb of clean data were obtained. The clean data of each sample reached 6.03 Gb, and the percentage of Q30 bases was 92.77% and above (Table S2).

**Figure 2:**
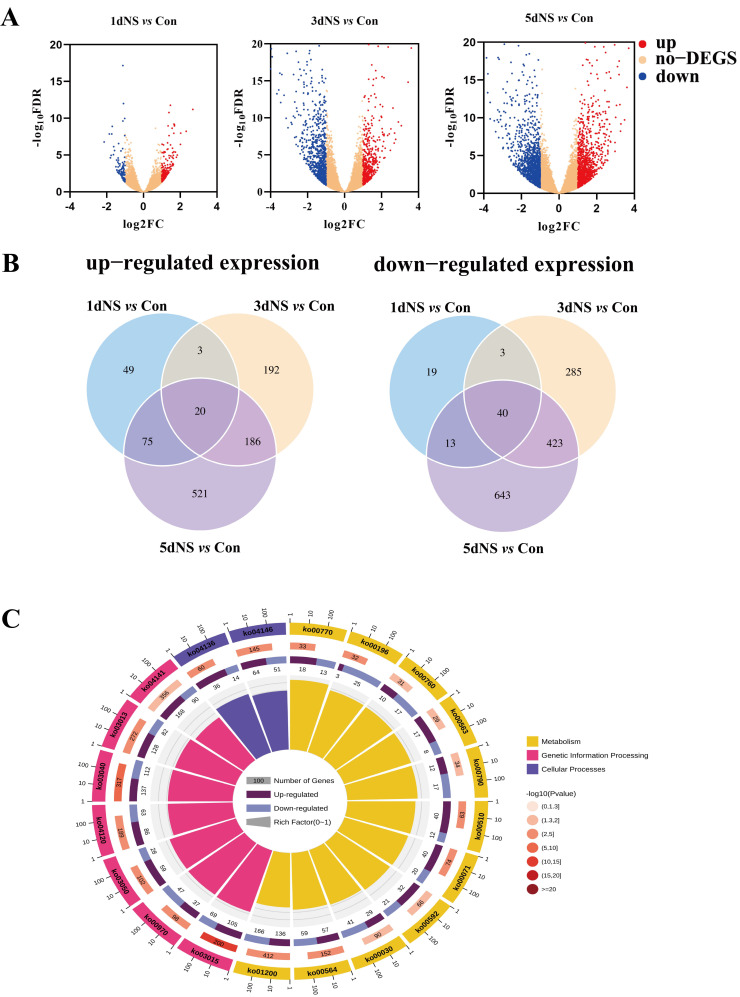
Transcriptome analysis of maize seedling leaves under phospholipase C inhibition. (**A**), volcano plot of differentially expressed genes (DEGs); (**B**), Venn diagram of DEGs; (**C**), 5dNS *vs.* Con Roundup of the top 20 significantly enriched differential pathways (yellow: metabolism; pink: genetic information processing; purple: cellular processes).

The distribution of total differentially expressed genes (DEGs) in the transcriptome can be seen in the volcano plot (Figure 2A). The largest number of differentially expressed genes was found in the “5dNS *vs.* Con” comparison group, which had 2664 DEGs, whereas in the “1dNS *vs.* Con” comparison group, the number of DEGs was relatively small.

The analysis of DEGs (|log_2_FC| ≥ 1) among the comparison groups showed that 147 genes were up-regulated and 75 genes were down-regulated in the “1dNS *vs.* Con” comparison group (Figure 2B). A total of 401 genes were up-regulated and 751 genes were down-regulated in the “3dNS *vs.* Con” comparison group. In the “5dNS *vs.* Con” comparison group, the number of up-regulated genes was 802 and the number of down-regulated genes was 1119.

The DEGs were annotated to the KEGG database, and the significantly enriched KEGG pathways were analyzed and demonstrated in Figure 1C. Under NS inhibitory conditions, DEGs were mainly enriched in peroxisome (ko04146), ubiquitin-mediated protein hydrolysis (ko04120), carbon metabolism (ko01200), glycerophospholipid metabolism (ko00564), pentose phosphate pathway (ko00030) and photosynthesis-antenna protein (ko00196) (Figure 2C and Figure S1). The “carbohydrate metabolism” contains the largest number of annotated genes (Figure S2). These data imply that inhibition of phospholipase C activity may affect cell membrane stability and photosynthesis, as well as carbon metabolism.

### 3.3. Inhibition of Phospholipase C Affects Photosynthesis in Maize Seedlings

**Figure 3:**
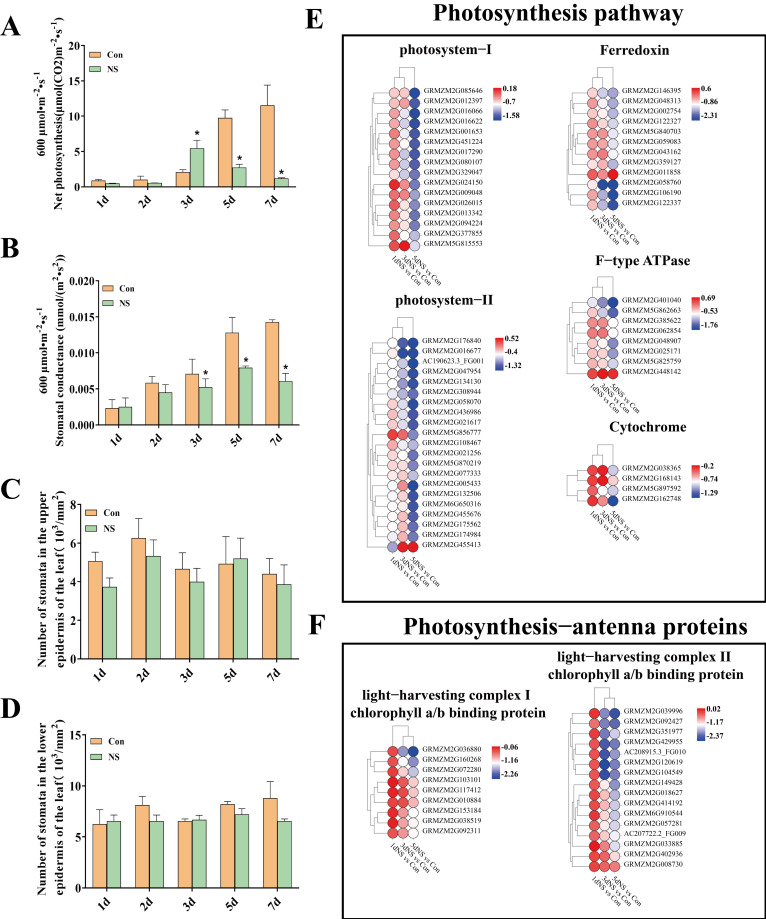
Changes in photosynthesis in maize seedling leaves under phospholipase C inhibition. (**A**), net photosynthesis; (**B**), stomatal conductance; (**C**), number of stomata in the upper epidermis of the leaf blade; (**D**), number of stomata in the lower epidermis of the leaf blade; (**E**), photosynthetic pathway (ko00195); (**F**), photosynthetic antenna proteins (ko00196). Values are shown as mean ± SD (*n* = 3 individuals per group). * means that the difference from the control reaches a significant level, *p* < 0.05.

Photosynthesis is a process in which green plants absorb solar energy, fix CO_2_, release O_2_ and synthesize organic matters. Under the light intensity of 600 μmol·m^−2^·s^−1^, the maximum net photosynthesis of NS samples reached 5.46 μmol·m^−2^·s^−1^ after 3 d (Figure 3A). The stomatal conductance peaked after 5 d (Figure 3B), even though there was no significant difference in the number of stomata (Figure 3C,D). The net photosynthetic parameters were also measured at different light intensities and time points, and it was apparent that the photosynthetic rate and dark respiration were decreased in the NS samples compared with the controls (Figure S3B).

The transcriptome data were screened and the DEGs involved in the photosynthesis processes (KEGG database ko00195 and ko00196) were recruited, and their expression profiles were depicted by heatmap icons (Figure 3E,F). The results revealed that the expression of DEGs involved in all processes related to photosynthesis, including photosystems and light-harvesting complexes, was obviously down-regulated under NS inhibition, especially at the 5-d time point.

### 3.4. Inhibition of Phospholipase C Affects Photosynthetic Pigment Accumulation in Maize Seedlings

**Figure 4:**
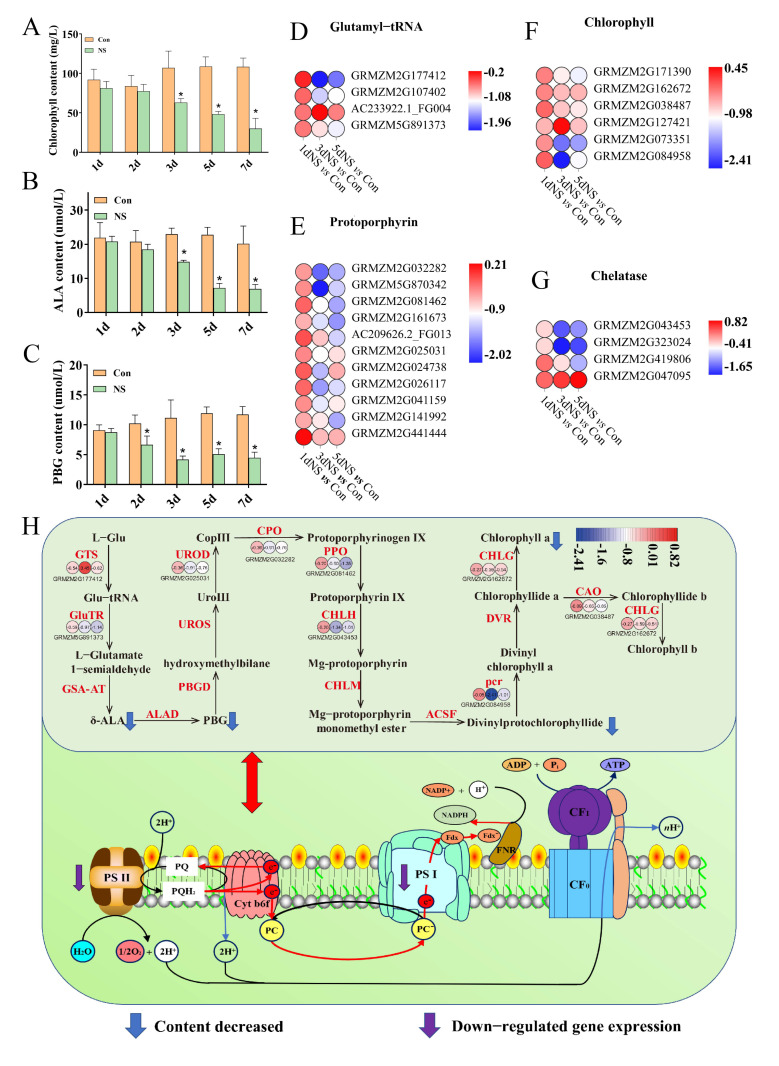
Changes in photosynthetic pigment synthesis in maize seedling leaves under phospholipase C inhibition. (**A**), chlorophyll content; (**B**), ALA content; (**C**), PBG content; (**D**), DEGs related to Glu-tRNA; (**E**), DEGs related to porphyrinogen; (**F**), DEGs related to chlorophyll; (**G**), DEGs related to chelatase; (**H**), chlorophyll synthesis pathway. The small circles represent the heat map of DEGs, and the numbers represent the fold change. Values are shown as mean ± SD (*n* = 3 individuals per group). * means that the difference from the control reaches a significant level, *p* < 0.05.

Chlorophyll is necessary for photosynthesis in green plants, and changes in chlorophyll content directly affect photosynthesis as well as the accumulation of photosynthetic products. In order to investigate the effect of phospholipase C inhibition on chlorophyll metabolism, the contents of chlorophyll, chlorophyll precursors and anthocyanins were measured. Under the 100-mM NS treatment, chlorophyll gradually decreased with increasing treatment time, and the difference reached a significant level after 3 d, whereas the chlorophyll content of the control remained at a relatively stable level (Figure 4A). The level of anthocyanins, on the contrary, gradually increased after 3 d of treatment, whereas the anthocyanin content of the control remained basically unchanged (Figure S4). The content of two chlorophyll precursors, α-aminolevulinic acid (ALA) and cholestyramine (PBG), were significantly lowered under NS treatment (Figure 4B,C).

From the RNA-seq data, 25 genes involved in chlorophyll metabolism were screened out. Four genes related to Glu-tRNA were down-regulated, among which GRMZM2G177412 showed the highest differential expression after 3 d, with a log_2_FC (3dNS *vs.* Con) of −1.96 (Figure 4D). All 11 genes related to porphyrinogen synthesis were down-regulated, especially in the “3dNS *vs.* Con” and “5dNS *vs.* Con” comparison groups (Figure 4E). Among these genes, the transcription of GRMZM5G870342 was most significantly down-regulated by 2.02-fold under 3 d of NS treatment. A total of six genes involved in chlorophyll synthesis were identified, two of which (GRMZM2G073351 and GRMZM2G084958) were drastically down-regulated after 3 d of NS treatment (Figure 4F). Four genes were found encoding chelatase, and three of them were obviously down-regulated under NS treatment (Figure 4G). In the chlorophyll synthesis pathway, the key genes involved in chlorophyll synthesis were down-regulated (Figure 4H), including GTS (GRMZM2G177412), GluTR (GRMZM5G891373), UROD (GRMZM2G025031), PPO (GRMZM2G081462), CHLH (GRMZM2G043453) and CAO (GRMZM2G038487).

### 3.5. Inhibition of Phospholipase C Affects Carbon Metabolism in Leaves of Maize Seedlings

**Figure 5:**
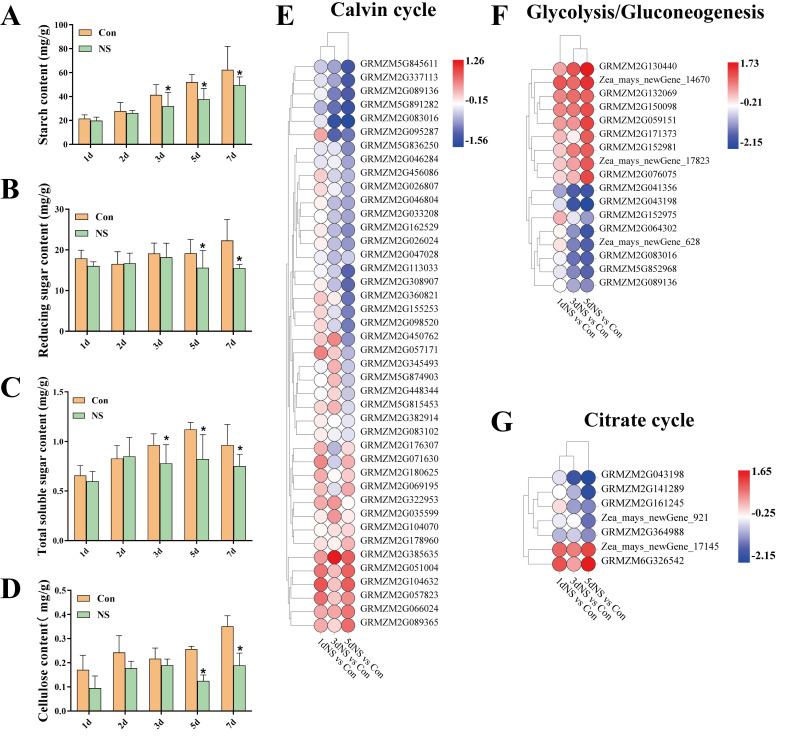
Analysis of carbon metabolism in maize seedling leaves under phospholipase C inhibition. (**A**), changes in reducing sugar content; (**B**), changes in starch content; (**C**), changes in total soluble sugar content; (**D**), changes in cellulose content; (**E**), Calvin cycle pa−1thway DEGs; (**F**), glycolysis/gluconeogenesis pathway DEGs; (**G**), tricarboxylic acid cycle pathway DEGs. Values are shown as mean ± SD (*n* = 3 individuals per group). * means that the difference from the control reaches a significant level, *p* < 0.05.

The contents of reducing sugars, total soluble sugars, starch and cellulose in the leaves of maize seedlings were measured, and the results are shown in Figure 5. The reducing sugar content did not show any significant changes in the first 3 days of NS treatment. However, at the 5-d time point, the reducing sugar content was significantly lowered in the NS samples compared to the control (Figure 5A). The starch content increased with the time of treatment, but the increase was lower than that of the control (Figure 5B). Total soluble sugar content of the NS samples increased and then decreased under NS treatment, reaching a maximum value of 0.85 mg/g at the 2-d time point, and then was significantly lowered compared to the control (Figure 5C). The level of cellulose was much lower than that of control, especially at the 5-d time point (Figure 5D).

From the RNA-seq data, 66 DEGs involved in four major carbon metabolic pathways were retrieved. Forty-two genes related to the Calvin cycle (ko00710) were identified, and more than half of them were down-regulated under NS treatment (Figure 5E). Four genes encoded PGK, three of which (GRMZM2G083016, GRMZM2G089136, GRMZM2G047028,) were significantly down-regulated at later stages of NS treatment. There were five genes encoding GAPD and all of them were down-regulated except GRMZM2G176307 and GRMZM2G180625. Seventeen DEGs were related to glycolysis/gluconeogenesis (ko00010) (Figure 5F). Among them, all genes related to the rate-limiting enzymes of glycolysis were down-regulated, including phosphoglycerate kinase (GRMZM2G083016, GRMZM2G089136) and enolase (GRMZM2G064302, Zea_mays_newGene_628). Seven DEGs were involved in the tricarboxylic acid cycle (ko00020), five of which were down-regulated (Figure 5G). These results suggest that the inhibition of phospholipase C affects the production and accumulation of photosynthetic products in the leaves of maize seedlings at the transcriptional level.

### 3.6. Inhibition of Phospholipase C Activity Affects the Stability of Cell Membranes

**Figure 6:**
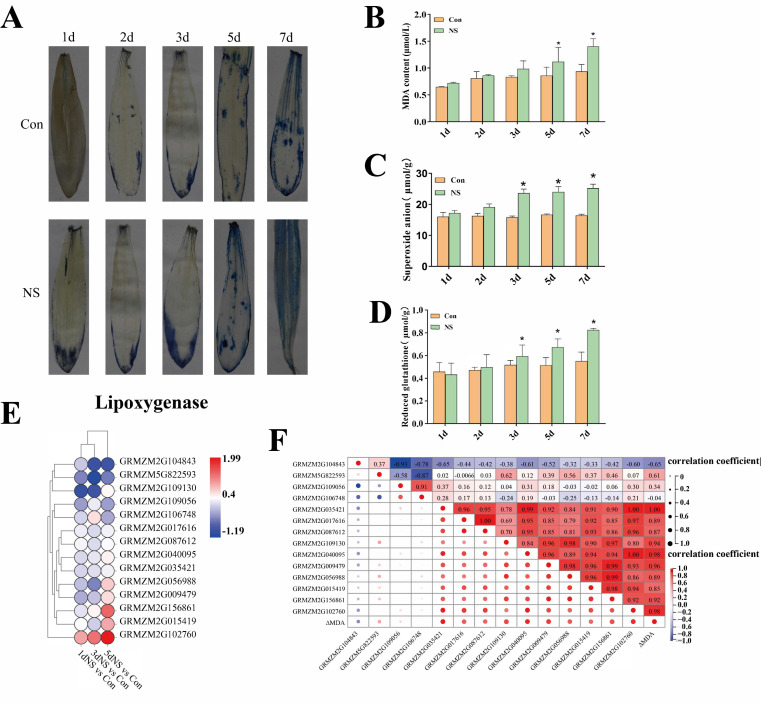
Effect of phospholipase C inhibition on cell membrane stability. (**A**), Evans blue staining; (**B**), changes in MDA content; (**C**), change in reduced glutathione content; (**D**), change in superoxide anion content; (**E**), LOX DEGs; (**F**), correlation analysis of LOX gene expression and changes in MAD content. Values are shown as mean ± SD (*n* = 3 individuals per group). * means that the difference from the control reaches a significant level, *p* < 0.05.

Evans blue staining was performed on leaves of maize seedlings under phospholipase C inhibition, and the results are shown in Figure 6A. The blueness of NS-treated leaves was deeper than that of the control group, indicating that the cell membrane was damaged more seriously. Moreover, the coloration gradually deepened with the increasing treatment time, suggesting increased damage under the phospholipase C inhibition.

To further determine the extent of cell damage in maize leaves under phospholipase C inhibition, we measured the malondialdehyde (MDA) content, and the results are shown in Figure 6B. At the early stage of NS treatment (1 d), the MDA content did not change significantly, whereas it gradually increased with treatment time. The difference between NS and Con reached a significant level after 5 d of treatment, and the maximum MDA content of the NS sample was 40 μ mol/L after 5 d. We also detected the changes of superoxide anion and reactive oxygen species scavenger (reduced glutathione) (Figure 6C,D). Under NS treatment, the level of superoxide anion (Figure 6C) and reduced glutathione (Figure 6D) consistently increased, whereas the con group remained at relatively stable levels.

Lipoxygenases (LOXs) are related to plant membrane lipid peroxidation and cell membrane stability. In this study, nine maize LOX encoding genes were detected in the transcriptome database, and their differential transcriptional expression is shown in Figure 6E. Most of the LOX genes were up-regulated under NS treatment in comparison to the control, among which GRMZM2G102760 (LOX5) represented the highest degree of up-regulation (Log2FC = 1.99) in the “5dNS *vs.* Con” group. The correlation of the expression of LOX genes and the content of MDA was analyzed (Figure 6F). For most of the LOX genes, a positive correlation was established between the differential expression of LOXs and the changes in MDA content, except for GRMZM2G104843 and GRMZM2G106748, which showed a negative correlation with the changes in MDA content (−0.65, −0.04). These indicate that the changes in MDA content might be caused by the up-regulated expression of LOX genes.

## 4. Discussion

The plasma membrane (PM) is a key structure that protects the cell, regulates nutrient exchange and allows the cell to sense signals [18]. The plasma membrane is mainly composed of phospholipids [19], phosphatidylinositol, sphingolipids [20,21] and sterols [22]. Phospholipids account for about 30% of PM lipids, phosphatidylcholine (PC) and phosphatidylethanolamine (PE) are the major phospholipids of the plant PM, and phosphatidylglycerol (PG), phosphatidylinositol (PI), phosphatidic acid (PA) and phosphatidylserine (PS) are the minor phospholipids [19,23,24]. Phospholipase C catalyzes the hydrolysis of phospholipids PC(PE) and PI, and plays important roles in plant growth and development [25].

In this study, 100 mM of neomycin sulfate (NS) was used as an inhibitor of phospholipase C activity and was applied to two-week-old maize seedlings. The results indicated that when phospholipase C activity was inhibited, it restrained the normal growth and development of maize seedlings, and affected the development of roots. In *Arabidopsis*, a similar phenomenon was observed when using the phospholipase C inhibitor [13]. Recently, PLC2 was found to regulate the polarity distribution of the growth hormone efflux carrier PIN2, thereby regulating growth hormone distribution and root growth and development in *Arabidopsis* [10]. The *Atplc2* mutants also exhibit shorter primary roots, impaired root gravity and impaired root hair growth [11].

A chloroplast is the site of light reaction and the electron transport chain in photosynthesis in higher plants. The lipid group of the chloroplast membrane is unique and mainly consists of uncharged monogalactosyl diacylglycerol (MGDG) and digalactosyl diacylglycerol (DGDG) [26,27]. It has been shown that when MGDG is lacking, the membrane energetics and photoprotection are impaired [28]; whereas the deficiency of DGDG leads to the dissociation of extrinsic proteins of the photosystem II (PSII) reaction center complex [29]. The photosynthetic electron transport chain is located in the vesicle-like membrane and consists of photosystem II (PSII), cytochrome (Cytb6f), photosystem I (PSI), plastid quinone (PQ) and plastid cyanidin (Pc) [27]. Previous studies suggested that, in 18:3 plant maize, the biosynthesis of MGDG and DGDG depends on phospholipase PLD- and PLC-mediated hydrolysis of phospholipids to provide the precursors of diacylglycerol (DAG) [14,30]. In this study, the photosynthesis and chlorophyll synthesis in maize leaves were both repressed under the treatment of the PLC inhibitor at both biochemical and transcriptional levels. We hypothesized that the stability of the chloroplast envelope might be affected by the inhibition of phospholipase C activity, owing to the disruption of DAG metabolic balance. When the stability of the chloroplast envelope is disrupted, the binding of chlorophyll to the thylakoid membrane is blocked, which might affect the chlorophyll synthesis and photosynthesis. The involvement of phospholipids in regulating photosynthetic electron transport activities was investigated by phospholipase C treatment of isolated vesicles [31]. The results showed that phospholipase C could hydrolyze as much as 40–50% of phospholipids in the chloroplast membrane, resulting in a dramatic decrease in electron transport in photosynthesis [31].

**Figure 7:**
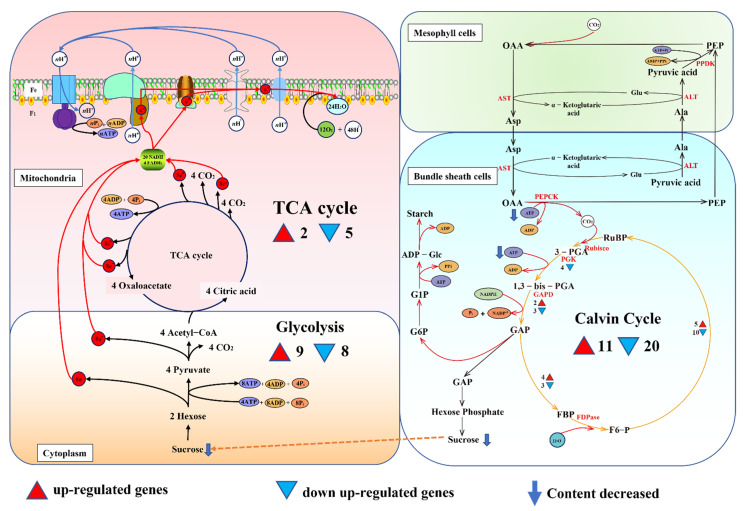
The carbon metabolism in maize seedling leaves under phospholipase C inhibition. (OAA: oxaloacetate; PEP: phosphoenolpyruvic acid; Asp: aspartic acid; Ala: alanine; PPDK: pyruvate phosphate dikinase; ALT: alanine aminotransferase; AST: aspartate aminotransferase; PEPCK: phosphoenolpyruvate carboxykinase; RuBP: ribulose diphosphate; Rubisco: ribulose diphosphate carboxylase; 3-PGA: 3-phosphoglyceric acid; 1,3-bis-PGA: 1,3-diphosphoglyceric acid; PGK: phosphoglycerate kinase; GAPD; glyceraldehyde-3-phosphate dehydrogenase; FDPaes: fructose 1,6-diphosphate phosphatase; GAP: glyceraldehyde triphosphate; FBP: fructose 1,6-bisphosphate; F6-P: fructose 6-phosphate; G6P: glucose 6-phosphate; G1P: glucose 1-phosphate).

The impact of phospholipase C inhibition on carbon metabolism in leaves of maize seedlings was remarkable in this study, and the main findings were summarized and illustrated (Figure 7). The processes of plant carbon metabolism include photosynthesis, photorespiration and respiration [32]. Maize is a typical C4 plant, in which CO_2_ is fixed in mesophyll cells and then transported to vascular bundle sheath cells for dark reactions, and are later transported to the vascular sheath cells for the dark reaction [33].

Respiration includes glycolysis, the tricarboxylic acid cycle (TCA cycle) and electron transfer/oxidative phosphorylation, with glycolysis occurring in the cytoplasm, the tricarboxylic acid cycle in the mitochondrial matrix and electron transfer/oxidative phosphorylation in the inner mitochondrial membrane [34].

It has been demonstrated that when the ratio of MGDG:DGDG increases, it induces overproduction of jasmonic acid, changes the shape of chloroplasts and inhibits photosynthesis [35]. In the present study, when phospholipase C activity is inhibited, DAG metabolism is affected, which could disrupt the MGDG:DGDG ratio in chloroplasts and result in a decrease in sugar production in photosynthesis. We found that the light reaction of photosynthesis was blocked when phospholipase C was inhibited, which might lead to a decrease in the supply of ATP and NADPH required for the dark reaction (Figure 7). The genes involved in the major steps of the Calvin cycle were screened from the transcriptome database, among which 11 genes were up-regulated and 20 genes were down-regulated. PGK catalyzes an important step in the glycolytic pathway of all organisms [36]. Studies have shown that ZmPGKs are involved in the photosynthetic metabolism in maize, and play a major role in photosynthetic metabolism [37]. We found that genes (GRMZM2G083016, GRMZM2G089136) encoding ZmPGKs were down-regulated under phospholipase C inhibition (Figure 7, Table S3). In the Calvin cycle, a decrease in GPA production may lead to a decrease in sucrose and starch synthesis, which is also consistent with the findings of this study.

In the glycolysis process, nine genes were up-regulated and eight genes were down-regulated (Figure 7). Pyruvate kinase (PK) is a key enzyme that catalyzes the final step of the glycolysis pathway, transferring the phosphate group of phosphoenolpyruvate (PEP) to ADP to produce ATP and pyruvate [38,39], and plays a crucial role in plant growth and development [34,40]. We found increased gene expression of pyruvate kinases, such as GRMZM2G171373 and GRMZM2G150098 (Table S3). This suggests that inhibition of phospholipase C activity has less effect on the glycolytic process.

In a mitochondrion, PC and PE are the major phospholipids, accounting for approximately 80% of total phospholipids [41,42]. When phospholipase C activity is inhibited, the metabolic balance between different lipids is disrupted, affecting mitochondrial membrane integrity and overall cellular function, including the TCA cycle. In the TCA cycle, seven DEGs were screened, two of which were up-regulated and five were down-regulated. Isocitrate dehydrogenase is considered a key rate-limiting enzyme in the TCA cycle and plays an important role in maintaining 2-ketoglutarate levels [43]. The expression of genes encoding isocitrate dehydrogenase (Zea_mays_newGene_921) and malate dehydrogenase (GRMZM2G141289) were down-regulated, which suggests that inhibition of phospholipase C has a great effect on the TCA cycle.

## 5. Conclusions

When phospholipase C was inhibited, the growth and development of maize seedlings were impaired. The phospholipase C inhibition significantly affected photosynthesis, photosynthetic pigment accumulation, carbon metabolism and the stability of the cell membrane in maize leaves. The differential expression of genes involved in the relevant metabolism pathways reflected the suppression of the chlorophyll synthesis pathway, light reaction, dark reaction and TCA pathway, which was consistent with the decreased photosynthesis and reduced photosynthetic products.

## Data Availability

The original data presented in this study are available in the Supplementary Materials. The RNA-seq data generated in this study were submitted to NCBI, under the accession number SUB9376981.

## Supplementary Materials

The following supporting information can be downloaded at: https://www.mdpi.com/article/10.3390/genes13061011/s1, Figure S1: circle diagram of the top 20 significantly enriched differential pathways. (**A**),1dNS *vs.* Con Roundup of the top 20 significantly enriched differential pathways; (**B**),3dNS *vs.* Con Roundup of the top 20 significantly enriched differential pathways (yellow: metabolism; pink: genetic information processing; purple: cellular processes). Figure S2: Statistical graph of KEGG path classification; Figure S3: Analysis of photosynthesis of maize seedling leaves under phospholipase C inhibition. A, stomatal observation; B, photosynthesis curve; Figure S4: Changes of anthocyanins in maize seedling leaves under phospholipase C inhibition; Table S1: Primer list for RT-PCR validation; Table S2: Sequencing data statistics; Table S3: Differential expression of genes related to carbon metabolism under phospholipase C inhibition.
