# Supplementary figures and images for: Involvement of Phospholipase C in Photosynthesis and Growth of Maize Seedlings
Source: Genes (Basel). 2022 Jun 3;13(6):1011. doi: 10.3390/genes13061011 (PMC9222606; doi:10.3390/genes13061011)

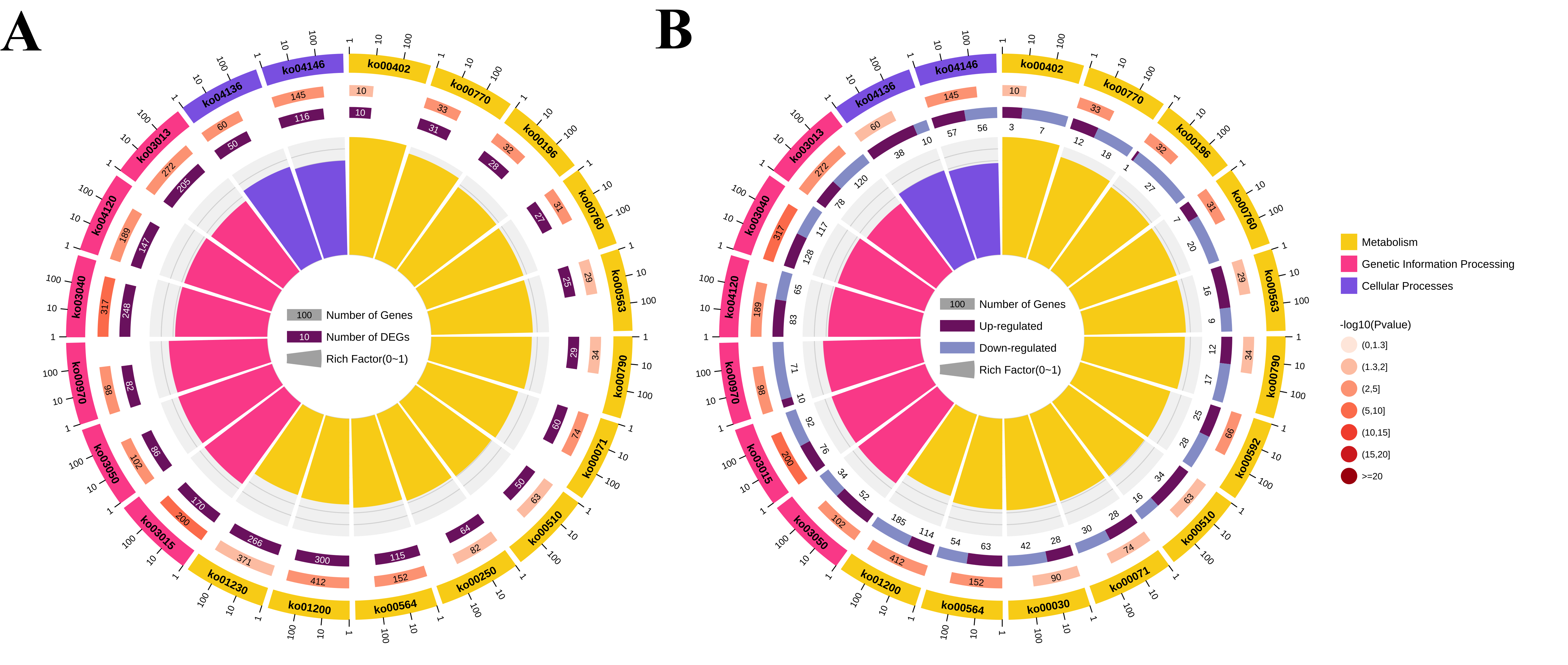

Supplement: Supplementary file 1 [file genes-13-01011-s001.zip › Figure S1.tif]

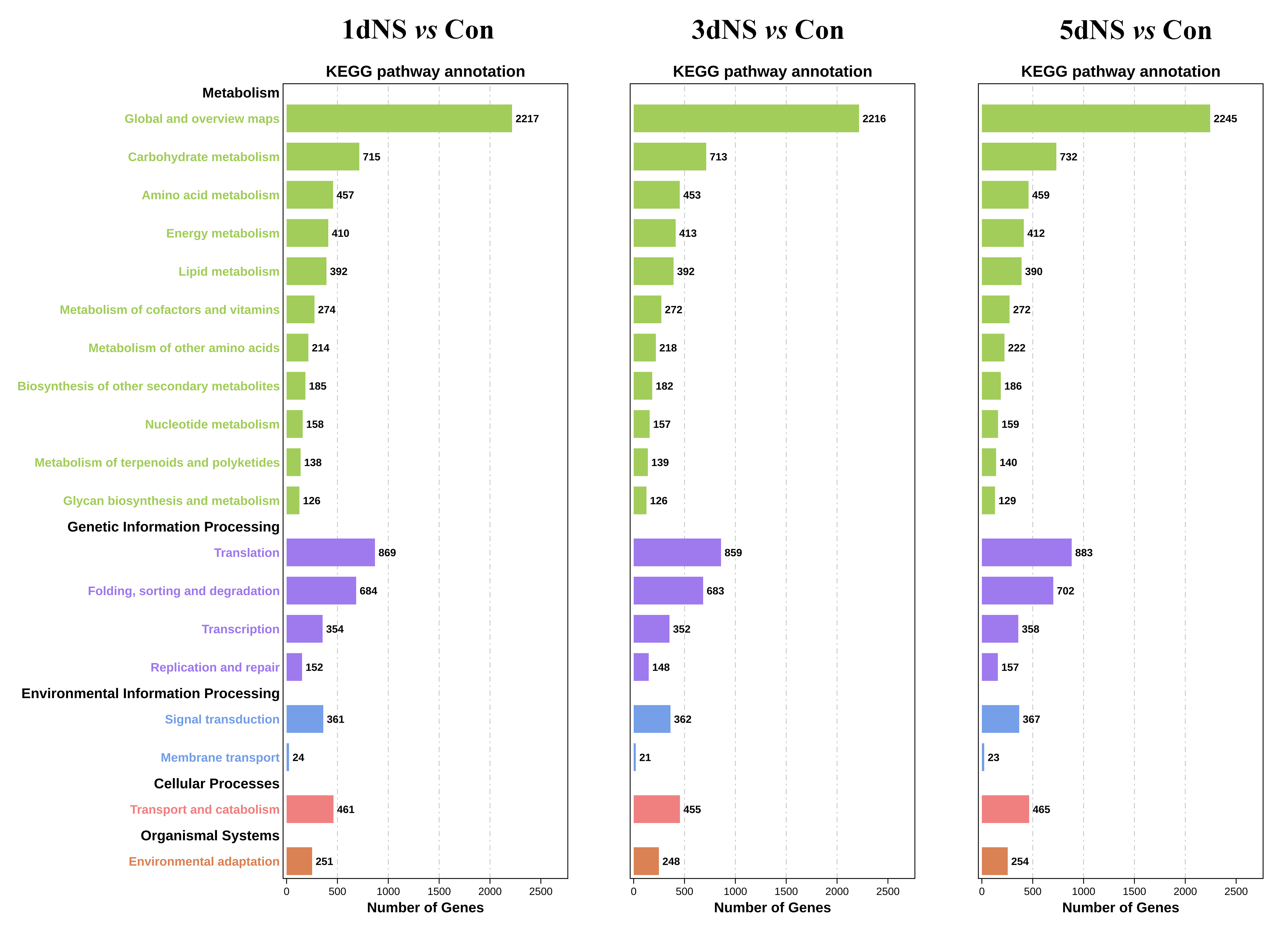

Supplement: Supplementary file 1 [file genes-13-01011-s001.zip › Figure S2.png]

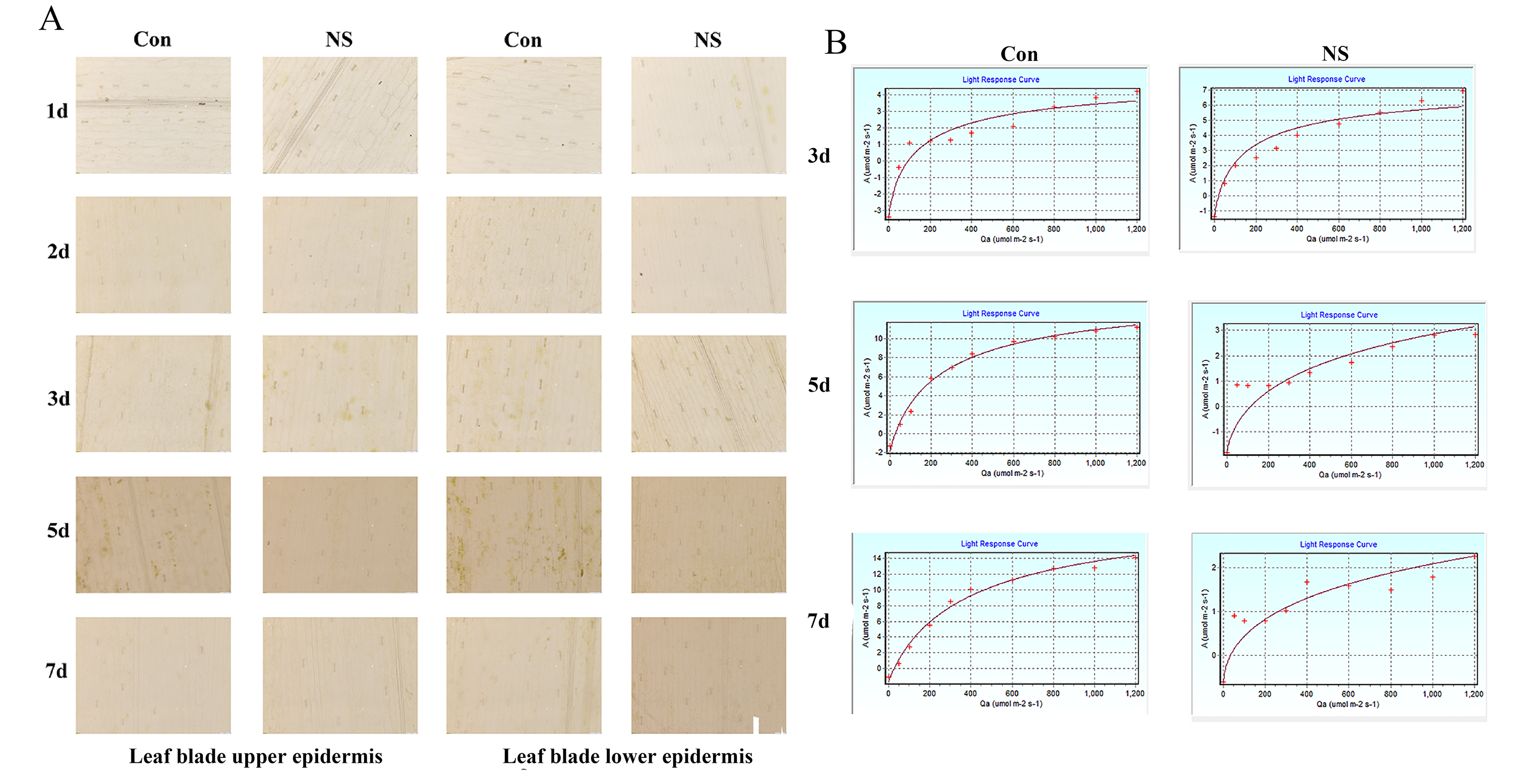

Supplement: Supplementary file 1 [file genes-13-01011-s001.zip › Figure S3.tif]

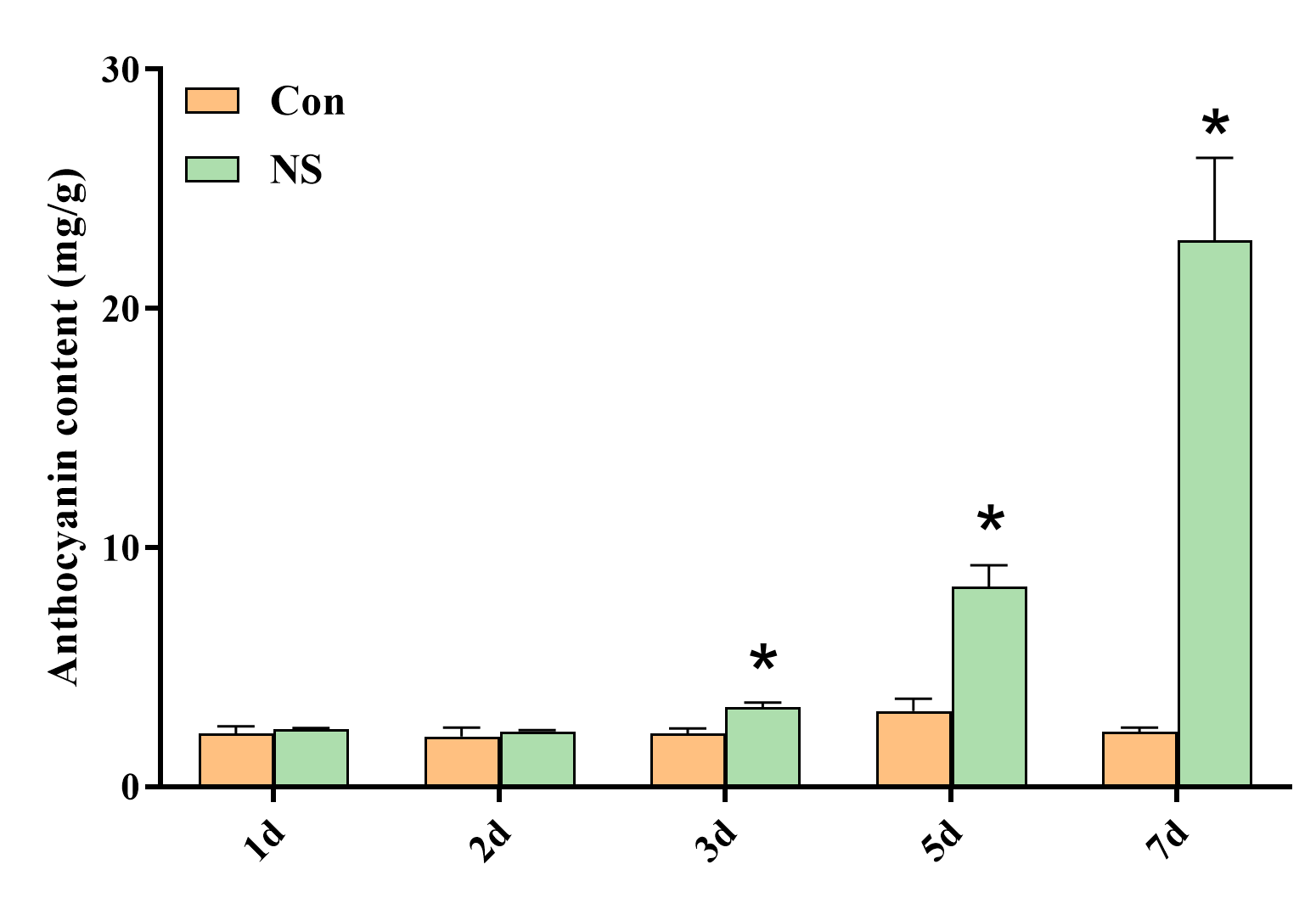

Supplement: Supplementary file 1 [file genes-13-01011-s001.zip › Figure S4.tif]
